# Community-based approach to detect and predict conflicts with large carnivores in human-dominated landscape

**DOI:** 10.1007/s13280-025-02241-6

**Published:** 2025-09-27

**Authors:** Izabela Fedyń, Marek Pasiniewicz, Katarzyna Zabiega, Hubert Fedyń, Michał Ciach

**Affiliations:** 1 Department of Forest Biodiversity, Faculty of Forestry, University of Agriculture in Krakow, al. 29 Listopada 46, 31-425 Krakow, Poland; 2 Bieszczadziki Foundation, ul. Bieszczadzka 106, 38-505 Bukowsko, Poland; 3 Regional Directorate for Environmental Protection in Rzeszów, ul. Józefa Piłsudskiego 38, 35-001 Rzeszów, Poland

**Keywords:** Adaptive management, Citizen science, Coexistence, Human-wildlife conflict, Stakeholder engagement

## Abstract

Understanding context-specific drivers and mechanisms of human-wildlife interactions is crucial to designing effective conservation strategies. Community-based data provides valuable insights for adaptive management efforts aimed at fostering coexistence in shared landscapes. This study analyzed 611 reports of grey wolves and brown bears recorded by rural communities in the Carpathians (Poland). Bears and wolves were recorded in 6% and 12% of all built-up areas within the study region, respectively. These species exploited anthropogenic food resources (53% of all reports), interacting with pets, livestock, and wild animals found within human settlements. The probability of carnivore reports increased with the number of previous observations and was influenced by prior successful food acquisition. The observed pattern highlights the importance of habituation and food conditioning for effective management of carnivores in human-dominated landscapes. Engaging local communities in monitoring carnivore presence can facilitate early conflict detection, providing a foundation for effective coexistence strategies.

**Supplementary Information:**

The online version contains supplementary material available at 10.1007/s13280-025-02241-6.

## Introduction

The distribution of wildlife is significantly influenced by human activities, which historically have led to spatial separation, local population declines, and in some cases, species extinction (Faurby and Svenning 2015; Bull and Maron 2016). Increasing awareness about the importance of preserving ecosystems and their functions has driven extensive efforts towards restoring species and habitats at scales ranging from local initiatives to global programmes (Malhi et al. 2016; Svenning et al. 2016; Perino et al. 2019). A flagship example of successful conservation measures is the return of megafauna, particularly grey wolves *Canis lupus* and brown bears *Ursus arctos*, to densely populated Europe, where they have significantly rebuilt populations within their historical ranges (Chapron et al. 2014; Boitani and Linnell 2015; Kaczensky 2024). Prolonged protection has ensured that wolf and bear are not classified as endangered at the European scale (Boitani 2018; Huber 2018), and their populations now occupy a wide range of multi-use landscapes (Cretois et al. 2021).

The return of large carnivores to European landscapes poses new conservation challenges (Boitani and Linnell 2015), as protected areas cover only 5–10% of their distribution ranges (Santini et al. 2016). Consequently, substantial carnivore populations reside in regions utilized by humans (López-Bao et al. 2017; Zanni et al. 2023) including urbanized ones (Morales-González et al. 2020; Cimatti et al. 2021). Wolves and bears adapt to anthropogenic areas by altering their temporal and spatial activity to minimize encounters with humans (Lodberg-Holm et al. 2019; Mancinelli et al. 2019; Carricondo-Sanchez et al. 2020). However, elements of the cultural landscape can be utilized by carnivores, such as military areas serving as ‘stepping stones’ for wolves colonizing western Europe (Reinhardt et al. 2019), or forest roads used by wolves to move efficiently across their home ranges (Bojarska et al. 2020). One of the examples of carnivore plasticity is the wolf’s expansion into areas larger than those initially identified in Poland as optimal habitat (Jedrzejewski et al. 2008; Gula et al. 2020).

Large carnivores provide essential ecosystem services and shape ecological relationships in the habitats they occupy (Kuijper et al. 2024). However, their presence in multi-use landscapes gives rise to diverse interactions that challenge coexistence between wildlife and humans, as well as among different groups of people (Peterson et al. 2010; Soga and Gaston 2022). Human–wildlife conflicts are inherently complex, varying in nature, intensity, and underlying causes across different contexts, involving multiple stakeholder groups with diverse values and perspectives (Zimmermann et al. 2020). At their core, they can be defined as ‘struggles that emerge when the presence or behaviour of wildlife poses actual or perceived, direct and recurring threats to human interests or needs, leading to disagreements between groups of people and negative impacts on people and/or wildlife’ (IUCN 2023). In the European context, where wolves and bears expand into areas where human activity is concentrated, concerns about livestock predation and public safety increase. Across their range, large carnivores are involved in conflicts with livestock husbandry (Kaczensky 1999; Blanco and Sundseth 2023; Cherepanyn et al. 2024). Mitigating these conflicts requires understanding the risk factors, implementing damage prevention methods, developing legal and financial mechanisms, and educating local communities (Linnell et al. 2012; Marucco and Boitani 2012; Bautista et al. 2017; Ostermann-Miyashita et al. 2025). As carnivore populations recover, new challenges emerge as these animals increasingly explore urban areas, seeking resources and inevitably interacting with people and their animals (Newsome et al. 2017; Kuijper et al. 2019). Direct interactions between carnivores and humans remain rare, suggesting that the threat these species pose to human health and life is low (Bombieri et al. 2019; Linnell et al. 2021). Nevertheless, the appearance of wolves and bears near human settlements generates considerable public and media attention (Chandelier et al. 2018; Neagu et al. 2022; Zscheischler and Friedrich 2022), while psychological cost associated with fear, perceived danger and risk shape public acceptance more than actual economic losses (Stăncioiu et al. 2019; Siemer et al. 2023). With growing populations of both people and carnivores, the likelihood of interactions will increase, emphasizing the need to develop strategies for coexistence in shared space (Carter and Linnell 2016; Chapron and López-Bao 2016; López-Bao et al. 2017). Repeated exposure to humans may lead to carnivores’ increased tolerance towards people (Wam et al. 2014), making it crucial to understand adaptation mechanisms and predict the consequences of carnivore presence near humans to protect their populations and minimize negative impacts on human well-being and livelihood (Carter and Linnell 2016). As Mech (2017) states, biologically, wolves will persist wherever humans tolerate them. Since conflicts often prompt lethal solutions targeting problem individuals or entire populations (Lamb et al. 2020), legislation and management will play critical roles in determining carnivore distribution through science-based strategies to mitigate human–wildlife conflict (Linnell et al. 2001; López-Bao et al. 2017).

Understanding conflicts at the human–wildlife interface and promoting coexistence are essential prerequisites for preserving nature in multi-use human-modified landscapes (König et al. 2020; IUCN 2023). Coexistence should be viewed as a dynamic process that considers local landscape specifics, communities, and carnivore populations (Ouvrier et al. 2025). Reliable data on conflicts are essential for effective solutions (Selva et al. 2023), and the experiences of local communities coexisting with large carnivores provide a foundation for designing conservation and conflict management strategies (Booth and Ryan 2019; Bonnet-Lebrun et al. 2020). Engaging the public through citizen or community science is increasingly recognized as an effective way to combine experiential and scientific knowledge (SSC 2023). Public participation has already been used to identify priority conservation areas for large carnivores and predict conflict-prone locations (Bonnet-Lebrun et al. 2020; Klees van Bommel et al. 2020). In this study, we demonstrate the potential value of local community experience in identifying and predicting conflicts with large carnivores in the rural Carpathians. The objectives of this study were to (1) identify the types of carnivore observations and associated food attractants reported by local community, (2) analyse landscape correlates of locations of these reports, and (3) assess the probability of subsequent reports based on the number and type of previous reports. We predicted that reports of carnivores in built-up areas would be mainly associated with food acquisition and that these reports would form local clusters driven by the presence of so-called problem individuals that repeatedly appear in human proximity and are engaged in behaviours such as searching for anthropogenic food, causing property damage, or preying on domestic animals (Majić Skrbinšek and Krofel 2015). We also expected that the increasing number of reports covering successful feeding events would raise the probability of future reports, driven by food conditioning.

## Materials and methods

### Study area

This study was conducted in southeastern Poland (Central Europe) within the administrative boundaries of 17 municipalities, covering a total area of 2793 km^2^. The entire study region is located in the Carpathian Mountains and encompasses mid- and low-mountain zones as well as foothills. The average forest cover across the studied municipalities was 56.1%, ranging from 13.1% to 90.1%. Due to the mountainous terrain, human settlements are primarily concentrated in valleys, resulting in low building density—an average of 51 buildings per km^2^ (ranging from 5.3 to 230 buildings per km^2^). The mean population density was 108 people per km^2^ (ranging from 5.9 to 876.8 people per km^2^). 13% of the population lives in urban areas, with the largest town in the study area being Sanok, which has approximately 37 000 inhabitants. The study region is characterized by a temperate climate with an average annual temperature of 10.4°C recorded in 2024. Seasonal variations in weather conditions include warm summers and cold winters. July is the warmest month with an average temperature of 20.2°C, while January is the coldest at − 0.7°C.

### Studied species

The study area is inhabited by Carpathian subpopulations of grey wolf and brown bear (Kaczensky et al. 2024), classified as least concern (Huber 2018; Boitani et al. 2022). Their ranges extend across the Carpathian mountain chain, including Serbia, Romania, Ukraine, Slovakia, Poland, Hungary, and the Czech Republic, and are considered among the largest carnivore populations in Europe (Kaczensky et al. 2024). Both wolf and bear are strictly protected under national law. Additional protection is provided through international legislation, including the Habitats Directive (wolf listed in Annexes II, IV, and V; bear in Annexes II and IV) and the Bern Convention. These legal frameworks protect live animals, body parts, and their habitats. Practically, they prohibit killing, disturbing, capturing, or destroying habitats, and all actions towards these protected species must not deteriorate their conservation status, assessed by population size and range. Exceptions, such as lethal control or capture, are only permitted through derogations to the strict protection regime that must be justified and targeted at specific individuals. Furthermore, signatories of the Habitats Directive are obliged to designate special conservation areas for wolves and bears and to regularly monitor and report on their conservation status. In the study area, Natura 2000 sites cover 38% of the territory, with wolves and bears listed as protected species. Monitoring data indicate that the population trend for both species in the region is increasing, while habitat quality and long-term viability are assessed as favourable. Identified threats to the long-term persistence of these species include infrastructure development and urbanization, availability of anthropogenic food, and negative public attitudes driven by damage caused by these species (European Commission 2019a, 2019b).

The national conflict management system includes compensation payments and permits for the removal of individual animals causing damage or posing a threat to human of livestock (e.g. in 2022 – 58 wolves, 0 bears). Compensation for economic losses (hereafter: damage) caused by protected species (such as damaged beehives or livestock losses) is provided by the state at market value. Damage verification and valuation are conducted by qualified inspectors from the Regional Directorates for Environmental Protection (RDEP), which are governmental agencies responsible for nature conservation at the regional level. However, encounters with large carnivores that do not result in damage, including visits to built-up areas, use of anthropogenic food are not officially monitored.

### Data collection

**Fig. 1:**
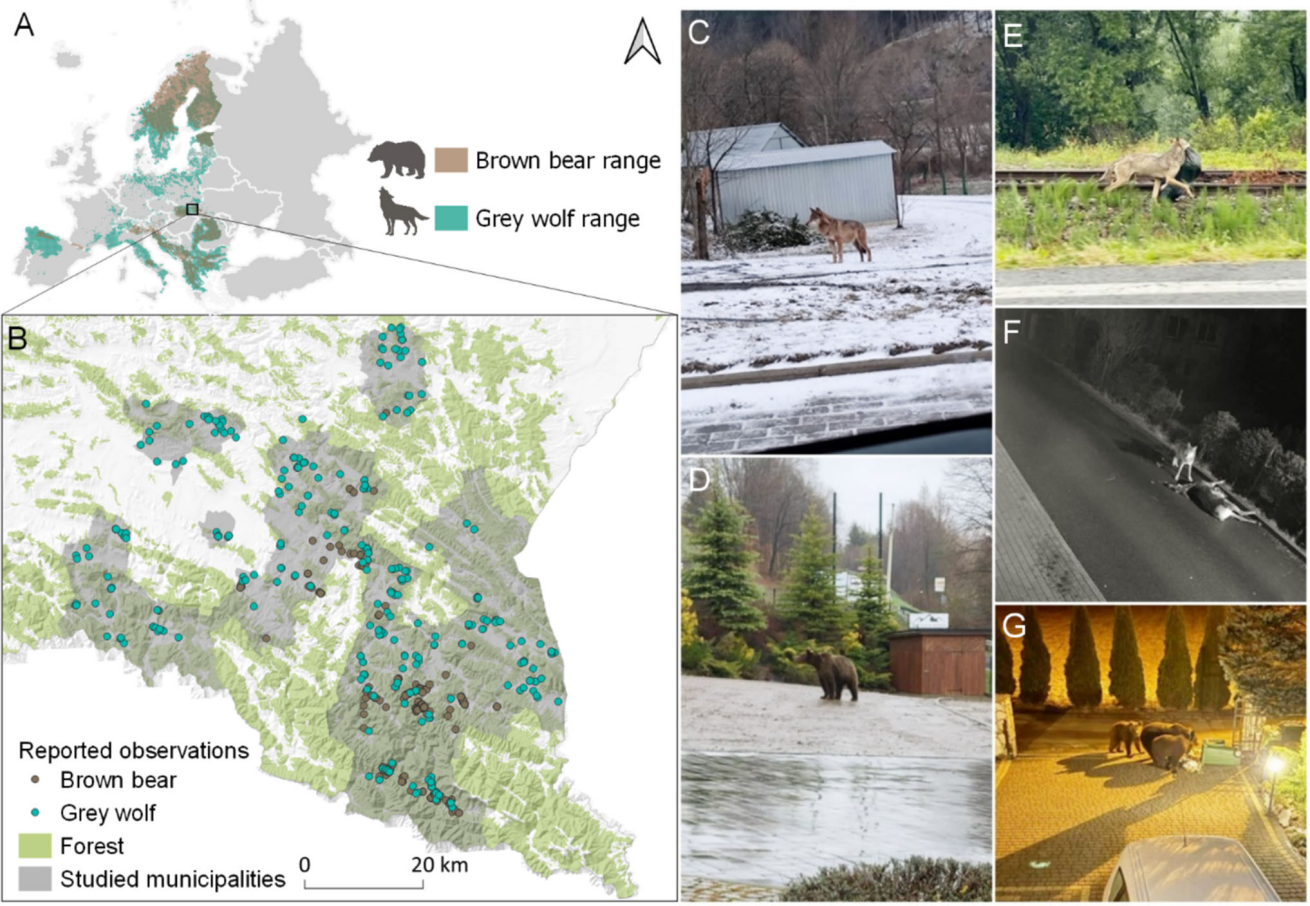
Study area and examples of reports of large carnivores recorded by local communities in the Carpathians, southeastern Poland. **A** Distribution range of brown bears *Ursus arctos* and grey wolves *Canis lupus* in Europe. **B** Reports of brown bear (N = 277) and grey wolf (N = 334) in the studied municipalities between 1st July of 2023 and 30th June of 2024. **C**–**G** Examples of reported observations of large carnivores near human settlements: (C) wolf near buildings (photo: Paweł Kaczmarczyk); (D) brown bear in a residential yard (photo: Robert Janczak); (E) wolf carrying a bag of waste along a road (photo: Ustrzyki Dolne Municipality Archives); (F) wolf with a hunted red deer *Cervus elaphus* in a built-up area (photo: Ustrzyki Cultural Center monitoring); (G) bears foraging on waste stored in household bins (photo: Jolanta Maszczak)

Reports of observations of wolves and bears in built-up areas were recorded using a dedicated form developed to monitoring by local communities encounters with large carnivores. The form was made available to the participating municipalities (Fig. 1) via KoboToolBox,^1^ a free open-source platform used to register reports of carnivore sightings. Data collection was conducted between 1st July of 2023 and 30th June of 2024. Representatives of the participating municipalities were introduced to the use of the form, and a designated person was appointed in each municipality to register reports. Submitted observations were verified through site inspections by trained municipal staff or RDEP personnel. Verification included searching for tracks, scats, and collecting supporting documentation such as photographs or surveillance footage. Each recorded observation was georeferenced and included the date and time. The observation point was defined as the exact location where the animal was seen or recorded by monitoring system. Based on interviews with the person reporting the observation and field verification, the presence of potential natural or anthropogenic food or social attractants (binary variable: present/absent; hereafter: attractants) was recorded within a 100 m radius of the observation point. The questionnaire included a list of potential attractants with multiple-choice options and an open field for unlisted entries (Appendix S1). All attractants reported were subsequently grouped into six general categories for analysis, which included livestock, beehives, fruit trees and shrubs, wild ungulates, dogs, and waste (Table 1). Based on the observed animal’s behaviour, each report was categorized into one of the following event types: (I) killing or injuring livestock or companion animals (hereafter: depredation on domestic animals), (II) damage to beehives, (III) property damage, (IV) foraging on anthropogenic food, (V) foraging on natural food, or (VI) other behaviours (movement, playing, or other unclassified activities). Anthropogenic food included waste, while natural food sources comprised wild ungulates and fruit trees or shrubs (Table 1). Additionally, if a food attractant was present, the outcome of the foraging attempt was recorded, i.e. whether the animal successfully accessed and consumed the food.

**Table 1:** Potential attractants within a 100 m buffer around the locations of reports of brown bears *Ursus arctos* (N = 277) and grey wolves *Canis lupus* (N = 334) recorded by local communities in the Carpathians, southeastern Poland

Investigated attractants	Description
Fruit trees or shrubs	Fruit-bearing trees and shrubs, primarily including apple *Malus* sp., plum *Prunus* sp., bilberry *Vaccinium* sp., blackberry *Rubus* sp.
Wild ungulates	Wild ungulate species present in the study area, either as live animals or carcasses: red deer *Cervus elaphus*, roe deer *Capreolus capreolus*, and wild boar *Sus scrofa*
Livestock	Live farm animals: cattle *Bos taurus*, horses *Equus caballus*, sheep *Ovis aries*, goats *Capra hircus*, farmed deers Cervidae, poultry including *Gallus* sp., *Anas* sp., *Columba* sp. and domestic rabbits *Oryctolagus cuniculus domesticus*
Dogs	Domestic dogs *Canis lupus familiaris*: house pets, free-ranging dogs, semi-feral and feral dogs
Beehives	Beehives inhabited by managed colonies of the honeybee *Apis mellifera*
Waste	Human-generated organic material stored as municipal waste (e.g. household bins, bags, composters)

### Data handling and analyses

Only data from municipalities that consistently recorded events throughout the study period were used for analysis. Of the 30 municipalities that joined the project, 18 maintained continuous records, collecting a total of 626 observations. For the purpose of this study, we assumed that repeated observations of the same individual within 100 m and a 6-h interval represented a single visit. Records were checked for duplicates, and observations from the same location and time were removed, resulting in a dataset of 611 unique observations. Statistical analyses were performed in RStudio (v. 2022.02.3), with significance set at *p* < 0.05. Spatial analyses were conducted in QGIS 3.22.8-Białowieża (QGIS Development Team 2020).

To characterize the location and circumstances of reports, distances were calculated from each observation point to the nearest building and to the forest edge as potential natural habitat. Additionally, the median number of buildings within a 100 m radius of bear and wolf locations were calculated. Data on building locations (including residential, industrial, and commercial structures) and forest cover were obtained from the national georeferenced vector database.^2^ Additionally, the proportion of daytime versus night visits and the proportion of visits involving human presence were calculated. Based on the observation time, visits were classified as daytime or nighttime according to sunrise and sunset times specific to each day. Human presence was noted if a person was outdoors and present at the start of the event. If a person was indoors or inside a vehicle, the event was classified as without human presence. To qualitatively describe large carnivore visits, the fraction of observations in each event type (described earlier in the *Data collection* section) was calculated along with associated attractants recorded within 100 m of the observation point.

For spatiotemporal analysis of the distribution of carnivore reports, the study area was divided into a grid of 0.01° cells (~ 1 km). The number of reports of wolves and bears was counted within each cell. Cells with less than 75% of their area within the administrative boundaries of the studied municipalities were excluded to avoid bias from unsampled regions. This resulted in 3294 grid cells covering the study area, including 1594 cells containing at least one building (hereafter: built-up areas). The percentages of grid cells with reports of wolves and bears were then calculated for each species. Only cells with at least one report of wolf or bear were used for further analysis. Spatial autocorrelation of report counts for each species was tested using Moran’s I. To assess the relationship between landscape features and number of reports in each grid cell, generalized linear models (GLMs) were constructed. The number of reports per species served as the response variable. Explanatory continuous variables included forest area and the number of buildings, while binary variables comprised: occurrence of foraging on anthropogenic food, depredation on domestic animals, damage to beehives (bear model), and killing wild ungulates (wolf model). Pearson correlations were checked prior to modelling. Due to low correlation values (*r* < 0.2), all variables were included. Overdispersion was assessed to determine the appropriate distribution, and final models were selected using backward selection to retain only significant variables.

To investigate the probability of subsequent reports based on previous number of reports, number of reports of wolves and bears were aggregated per grid cell in 30-day time windows. For each cell and time window, it was recorded whether a subsequent record occurred in the following 30-day period (1 = record reported, 0 = no report). Additionally, to evaluate whether the probability of subsequent reports varied by event type, the number of previous reports involving foraging or damage was also counted per cell and time window. Three binomial models were then constructed for both wolf and bear, modelling the probability of subsequent reports based on: (i) the total number of previous reports, (ii) the number of reports involving foraging, and (iii) the number of reports resulting in damage. Spatial logistic models from the spaMM package were used, incorporating geographic coordinates as a random effect (Matern function) to account for spatial autocorrelation between neighbouring cells (Rousset and Ferdy 2014). Model performance was evaluated using the Akaike Information Criterion (AICc), and the significance of explanatory variables was assessed based on p-values. Spatial correlation parameters were also calculated (*rho* indicating the spatial scale of correlation and *lambda* representing the variance of the spatial effect). The probability of subsequent reports relative to the number of previous reports of each type was computed based on model intercepts and regression coefficients.

## Results

### Characteristics of reports

During the study period, local communities of 18 monitored municipalities located in the Carpathians in southeastern Poland reported a total of 611 reports of large carnivores of which 277 involved bears and 334 involved wolves. The median distance between bear report locations and the nearest building was 18.6 m (IQR 9.9–40.2; range 0.5–1051.4), while for wolves it was 22.4 m (IQR 10.1–49.3; range 0.3–1631.7). The median number of buildings within a 100 m radius of location of reports of bears was 7 (IQR 3–12; range 0–34) and 7 for wolves (IQR 3–12; range 0–41). The median distance to the forest was 183.5 m for location of reports of bears (IQR 43.5–338.6; range 0–1142.5) and 305.9 m for wolves (IQR 136.8–547.8; range 0–3186.7).

**Fig. 2:**
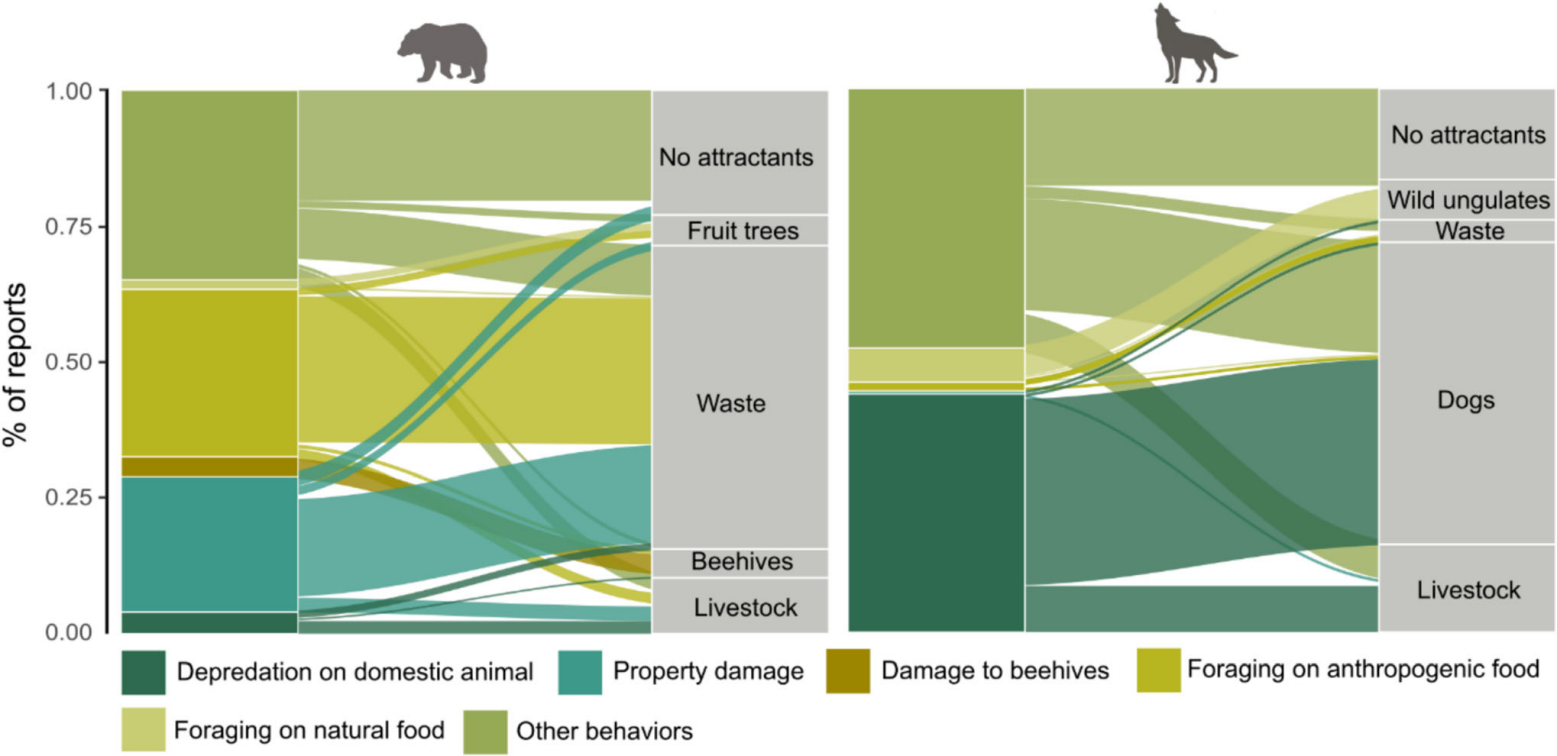
Types of events and attractants within a 100 m buffer around the locations of reports of brown bears *Ursus arctos* (N = 277) and grey wolves *Canis lupus* (N = 334) recorded by local communities in the Carpathians, southeastern Poland

Of the reports of wolves, 52.1% occurred during the day, while 69.3% of reports of bears took place in daylight (Supplementary Fig. S1). Human was absent during 79.9% of reports of wolves and 88.1% of reports of bears. Most reports of large carnivores were associated with foraging (Fig. 2). Bears successfully accessed food in 57.8% of reports, while wolves did so in 49.7%. The main attractants associated with carnivore reports were anthropogenic food sources such as waste, livestock, dogs, or beehives (Fig. 2). Waste was present in 60.6% of locations of reports of bears, while dogs were present in 60.8% of locations of reports of wolves. Wolves and bears also obtained natural food in build-up areas, such as wild ungulates or fruits (Fig. 2). Damage caused by large carnivore activity were recorded in 32.8% of reports of bears (including depredation on domestic animals, property damage, or damage to beehives) and in 45.7% of reports of wolves (mostly involving the depredation on domestic animals). A substantial proportion of reports (36.7% for bears and 46.3% for wolves) involved other behaviours such as movement or social interactions, and in approximately 22.0% of bear reports and 18.3% of wolf reports, no attractants were found (Fig. 2).

### Spatial pattern and landscape correlates of the number of reports

**Fig. 3:**
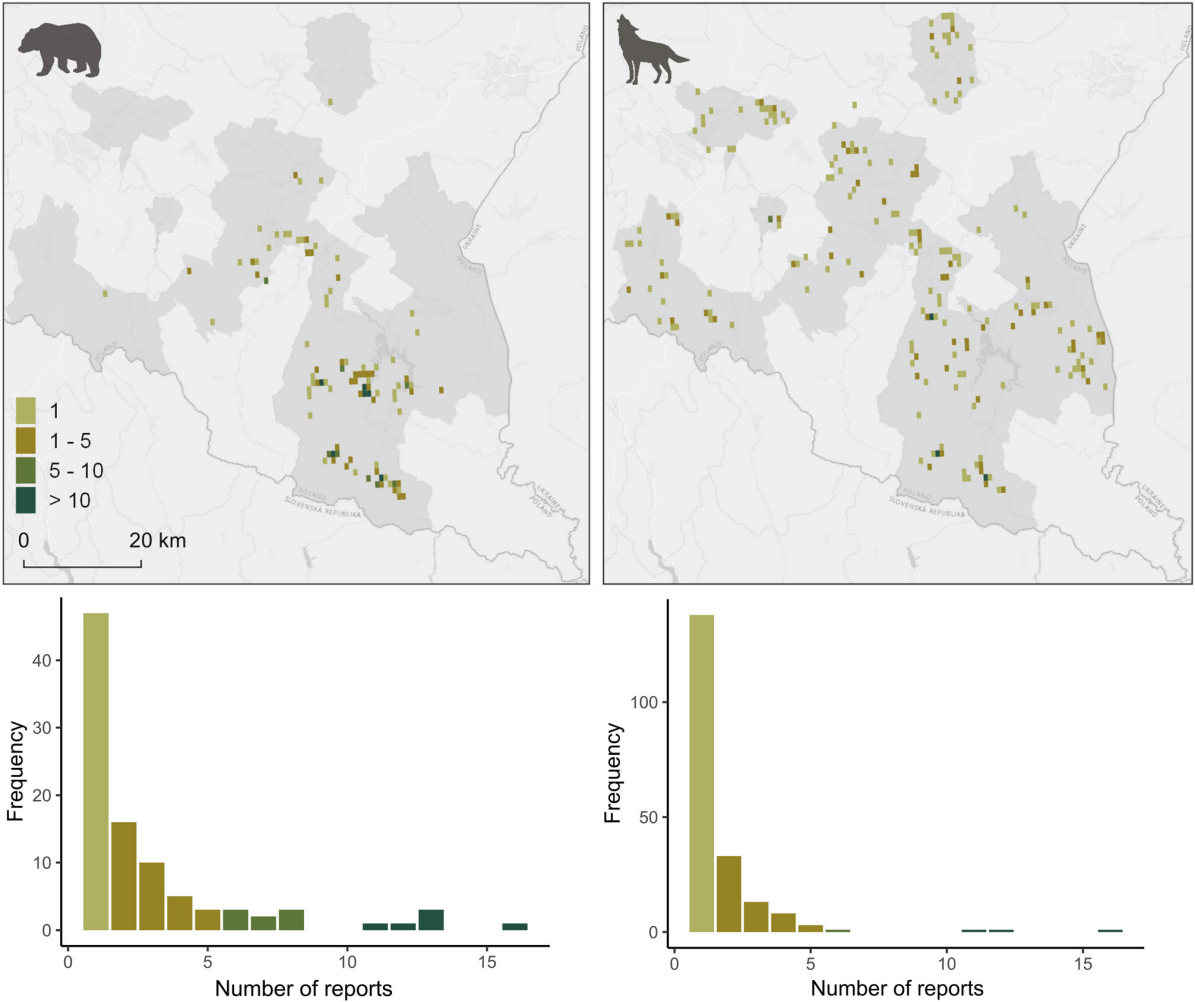
Number of reports of brown bears *Ursus arctos* (N = 277) and grey wolves *Canis lupus* (N = 334) within 0.01° × 0.01° grid recorded by local communities in the Carpathians, southeastern Poland

Throughout the study period, reports of bears were recorded in 2.9% of grid cells of the study area, while reports of wolves occurred in 6.0% (Fig. 3). When considering only built-up areas, reports of bears were recorded in 6.0% of these areas, and reports of wolves in 12.5%. Multiple reports were recorded in 50.5% of grid cells of the study area where bears were detected, with a mean of 2.9 reports per occupied cell (Fig. 3). The number of reports of bears per grid cell of the study area was positively associated with foraging on anthropogenic food and depredation on domestic animals (Table 2). The number of reports of bears was negatively correlated with forest cover within a grid cell (Table 2). Additionally, spatial autocorrelation of reports of bears was detected between neighbouring cells (Moran’s I statistic standard deviate = 1.92, *p* = 0.027). For wolves, 69.3% of grid cells with recorded species contained a single report, with an average of 1.7 reports per occupied cell (Fig. 3). No spatial autocorrelation was detected between neighbouring grid cells with records of wolves (Moran’s I statistic standard deviate = 1.17, *p* = 0.121). The number of records of wolves was positively correlated with the number of buildings and with depredation on domestic animals (Table 2).

**Table 2:** GLM showing the relationship between the number of reports of brown bears *Ursus arctos* (N = 95) and grey wolves *Canis lupus* (N = 199) and forest cover, number of buildings, and event type per 0.01° × 0.01° grid cell recorded by local communities in the Carpathians, southeastern Poland

		Starting model				Final model			
		Estimate	SE	t	p	Estimate	SE	t	p
Brown bear	Intercept	0.97	0.31	3.12	0.002	0.94	0.21	4.52	0.000
	Forest cover	− 0.10	0.06	− 1.77	0.079	− 0.10	0.05	− 2.00	0.048
	Number of buildings	0.00	0.00	− 0.11	0.913				
	Feeding on anthropogenic food	0.81	0.21	3.96	0.000	0.82	0.19	4.23	0.000
	Damage to beehives	− 0.01	0.46	− 0.03	0.976				
	Killing domestic animal	0.55	0.28	1.99	0.049	0.56	0.27	2.07	0.042
Grey wolf	Intercept	0.03	0.18	0.15	0.878	0.14	0.11	1.36	0.174
	Forest cover	0.02	0.03	0.44	0.659				
	Number of buildings	0.00	0.00	4.38	0.000	0.00	0.00	4.78	0.000
	Feeding on anthropogenic food	0.57	0.31	1.84	0.066				
	Killing wild ungulate	0.19	0.20	0.96	0.338				
	Killing domestic animal	0.31	0.12	2.55	0.011	0.26	0.11	2.31	0.021

### Probability of subsequent report

**Fig. 4:**
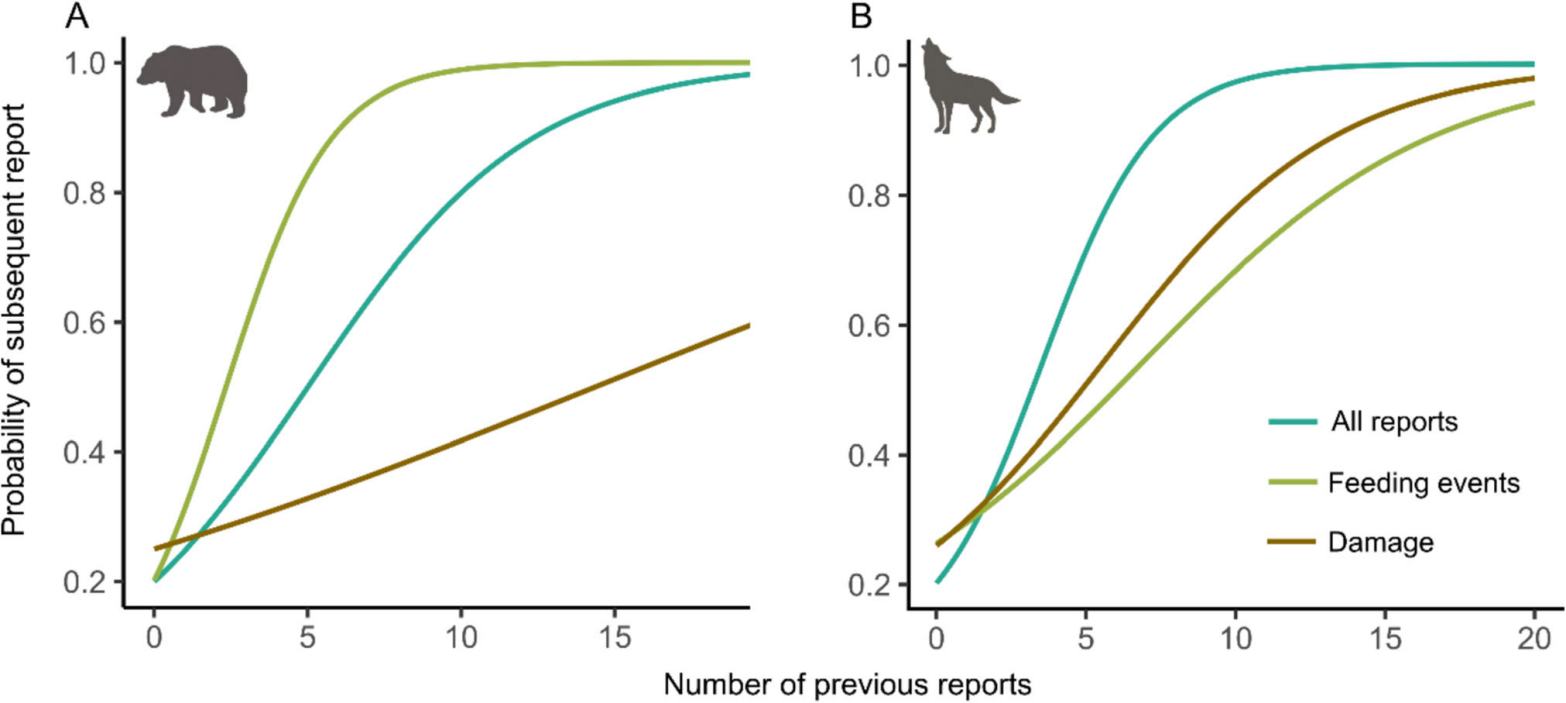
Probability of subsequent report of **A** brown bears *Ursus arctos* and **B** grey wolves *Canis lupus* as a function of the number of previous reports within a 0.01° × 0.01° grid over 30-day time windows recorded by local communities in the Carpathians, southeastern Poland. Probabilities are derived from spatial GLMMs (see Table 3)

The probability of subsequent report within a grid cell increased with the number of previous reports in the preceding time window for both bears and wolves (Fig. 4, Table 3). For bears, the probability of subsequent report also increased with the number of previous reports involving feeding (Table 3).

**Table 3:** Spatial GLMMs describing the relationship between the probability of subsequent reports of brown bears *Ursus arctos* and grey wolves *Canis lupus* and the number of previous reports, feeding events, and damage within 0.01° × 0.01° grid cells over 30-day time windows recorded by local communities in the Carpathians, southeastern Poland

Species	Variable	Fixed effects				Random effects			AICc
		Estimate	SE	z	p	rho	lambda		
Brown bear	Intercept	− 1.39	0.47	− 2.93	0.003	26.86	0.29		190.9
	Number of all reports	0.28	0.12	2.37	0.018				
	Intercept	− 1.37	0.39	− 3.56	0.000	36.68	0.22		178.6
	Number of feeding events	0.59	0.18	3.34	0.001				
	Intercept	− 1.10	0.53	− 2.08	0.038	26.08	0.47		196.2
	Number of damage	0.08	0.15	0.51	0.611				
Grey wolf	Intercept	− 1.91	0.36	− 5.24	0.000	21.47	0.10		288.95
	Number of all reports	0.54	0.21	2.56	0.010				
	Intercept	− 1.41	0.41	− 3.47	0.001	15.39	0.27		294.63
	Number of feeding events	0.20	0.20	1.02	0.308				
	Intercept	− 1.44	0.40	− 3.62	0.000	15.92	0.26		293.98
	Number of damage	0.26	0.19	1.33	0.185				

## Discussion

Based on reports submitted by local communities from areas inhabited by protected wolf and bear populations, we demonstrated the usefulness of residents’ experience in identifying and predicting locations of presumable conflicts. The reports covered a wide spectrum of interactions, ranging from economic losses, foraging on anthropogenic and natural food resources, and observations of animals moving through the area, reflecting the complex nature of large carnivore presence in built-up areas. The analysis revealed correlations between the number of reports of large carnivores, the availability of potential food sources and landscape characteristics, highlighting the importance of anthropogenic food as a driver of wolf and bear presence in build-up areas. Our results indicate that successful food acquisition significantly increased the probability of subsequent report in the same location, emphasizing the role of food conditioning in shaping the spatial patterns of large carnivore presence in human-modified landscapes. Nevertheless, the number of reports unrelated to foraging indicates that other ecological, behavioural, or social factors may also influence carnivore visits into human-dominated spaces. These findings emphasize that effective management strategies should focus foremost on removing attractants, while considering the broad context of human-carnivore interactions.

The global increase in human-provided food resources has led to a growing share of waste, crops, and livestock in large carnivore diets (Newsome et al. 2015). Anthropogenic food represents a spatially and temporally predictable resource offering high energetic gains at low foraging costs, making it highly attractive for opportunists (Schoener 1971). In our study, most locations from where large carnivores were reported contained anthropogenic food and significant proportion of these encounters was not associated with economic losses. Previously, research on human–large carnivore conflicts in this region focused on wolf attacks on livestock (Gula 2008; Fedyń et al. 2022), or bear damage to apiaries (Bautista et al. 2021; Berezowska-Cnota et al. 2023). This reporting bias stems from the official conflict reporting system in Poland, which documents only cases involving economic loss. As a result, management actions have historically focused on preventing damage to livestock, while interactions related to anthropogenic food sources or non-damaging carnivore presence have been largely overlooked, leaving a gap in coexistence strategies. Although preventive methods to limit large carnivore access to livestock or beehives are increasingly used in Europe, only a few coexistence projects have addressed the issue of preventing access to anthropogenic food sources or applying aversive interventions (Oliveira et al. 2021).

Our research shows that large carnivores are engaged in various interactions with other animals in human-modified landscapes, including wild ungulates that possibly venture into urbanized areas as a strategy to minimize predation risk (Muhly et al., 2011). At the same time, reports on interactions between wolves and domestic dogs are increasing, with dog-related events representing the majority of wolf observations in our study. Wolves kill dogs due to competition or opportunistic predation, but the close phylogenetic relationship between these species also creates potential for social interactions (Lescureux and Linnell 2014). Wolf–dog hybridization is commonly recorded in human-dominated landscapes and poses a serious conservation concern (Donfrancesco et al. 2019; Salvatori et al. 2019). We documented wolf visits that did not involve attacks on dogs, suggesting the potential for social interactions. Although the likelihood of wolf–dog interactions generally increases with distance from human settlements when dogs accompany humans during recreational activities within wolf ranges (Haidt et al. 2021), our findings show that such interactions also occur in built-up areas. Given that dogs are among the most common companion animals, there is an urgent need to minimize the probability of interface these animals (Donfrancesco et al. 2019).

The probability model developed in this study showed that repeated observations of carnivores increased the likelihood of subsequent report. Differences in the probability curves for wolves and bears may reflect distinct foraging strategies and the spatial distribution of attractants. In the case of wolves, which primarily preyed on dogs, the probability of subsequent report increased more slowly than for bears. This may be due to the scattered distribution of potential prey and the challenges associated with capturing it, unlike waste, which is widely available in urbanized areas. Additionally, bears’ manual dexterity allows them to actively access food, such as breaking into secured attractants (Dai et al. 2020), which is reflected by reported property damage associated with reports of bears. Furthermore, the number of bear reports was negatively correlated with forest cover and showed spatial autocorrelation, potentially indicating the presence of food-conditioned individuals exhibiting reduced movements and confined ranges within built-up areas (Cozzi et al. 2016). A key step in minimizing food conditioning is to prevent access to waste, fruit, and other potential resources (Crevier et al. 2021), which has proven effective in reducing conflict rates (Johnson et al. 2018). Failure to implement preventive measures allows carnivores to continue exploiting anthropogenic food, leading to conflict escalation and, in some cases, problem behaviour such as approaching humans or searching for anthropogenic food sources (Krofel et al. 2020; Reinhardt et al. 2020; Linnell et al. 2021).

Carnivore populations represent a personality spectrum, including individuals that learn faster and exhibit higher tolerance towards humans, making them more prone to exploring urbanized areas (Wam et al. 2014). Despite the presumed presence of problem individuals reflected by clusters of reports, most locations in our study contain single reports, which may suggest ongoing population-level adaptation to anthropogenic environments. In areas where human presence is predictable and carnivores are repeatedly exposed to humans without negative consequences, spatial segregation may gradually weaken (Blumstein 2016; Penteriani et al. 2017). While our study focused primarily on food attractants and interactions with domestic animals, a remarkable proportion of observations did not involve foraging behaviour, suggesting other possible motivations for entering built-up areas. Documented behavioural adaptations in anthropogenic landscapes include wolves using haystacks as shelter (Bojarska et al. 2024), while despotic distribution patterns may drive bears into urban spaces as a refuge from competition (Elfström et al. 2014; Mills et al. 2023). Although food conditioning is undoubtedly one of the key factors attracting carnivores and maintaining their presence in proximity to humans, the underlying drivers of adaptations to human-modified landscapes are likely multifaceted, involving trade-offs between risk, resource availability, and interspecific or intraspecific interactions. As occasional appearances of wolves and bears near human settlements are an inevitable element of coexistence in Europe, monitoring of all interactions and their drivers is crucial for managing potential conflicts, but also for ensuring the conservation of these species. Human-provided food subsidies alter life traits, including space use, behaviour and reproduction (Oro et al. 2013). In the long term, anthropogenic resources may act as a selective pressure influencing species’ evolutionary trajectories and creating ecological traps that increase human-caused mortality, with consequences at both individual and population levels (Newsome et al. 2015; Penteriani et al. 2018).

The community-based reporting method presented in our study can support proactive mitigation efforts, applied in areas with potential future conflicts. Stakeholder involvement and monitoring protocols are essential for adaptive management based on iterative development of action algorithms and assessment of effectiveness tailored to site-specific conditions (Williams and Brown 2014). Although community science is increasingly applied in conservation, such studies have limitations regarding data quality and interpretability. Data collected by non-specialists may include species misidentifications or misinterpretation of events. Moreover, reporting probability may depend on individual perceptions, experiences, and beliefs, leading to selective reporting and potential distortion of the actual conflict scale (Wilbur et al. 2018). Consequently, spatial differences in reporting may reflect social factors such as environmental awareness, local media narratives, or political agendas. This highlights the importance of verifying reports and educating local communities to raise awareness, particularly because situations without economic loss are rarely reported due to their perceived low importance (Wilbur et al. 2018). Early detection of the activity of large carnivore in built-up area is critical for conflict mitigation, as it enables timely, targeted measures to eliminate attractants and apply aversive conditioning to animals before undesired behaviours become habitual. At the same time, the widespread presence of certain food or social attractants, such as waste or domestic animals, presents a significant challenge. This indicates that effective strategy requires not only site-specific actions but also broader, system-level solutions incorporated into local policies and urban planning to reduce attractant availability across human-modified landscapes. A barrier to implementing preventive measures is cost of such measures (Noel and Pienaar 2017), which is why projects involving public participation should not only encourage engagement, but also set a positive example through responsiveness and meaningful action, ensuring long-term commitment. Procedural fairness and the competence of science and governance are key to building stakeholder acceptance and trust (Riley et al. 2018; Siemer et al. 2023). Sustainable human–carnivore coexistence requires integrating science, policy, and stakeholders to develop strategies that balance human needs with wildlife conservation (Ostermann-Miyashita et al. 2025).

## Conclusion

In this study, we characterized large carnivore observations based on reports from local communities living in areas where wolves and bears have been strictly protected for decades. Human presence is a permanent element of ecosystems colonized by large carnivores, offering both potential food resources and a context for interactions. Carnivore visits vary in nature and involve different types of attractants, most commonly resources widely available in built-up areas, such as waste, livestock, and pets. Although not all visits result in economic losses, the increasing number of visits raises the likelihood of further occurrences. This highlights the need for early conflict detection and prompt responses after the first incidents to prevent the reinforcement of problematic behaviours and conflict escalation. Monitoring areas where humans and carnivores overlap allows for identifying interfaces of interaction, which is crucial for developing effective coexistence strategies tailored to specific local contexts.

## Supplementary Information

Below is the link to the electronic supplementary material.

Supplementary file1 (PDF 671 KB)

## Data Availability

Data are available at 10.5281/zenodo.15068183.

## Footnotes

1 https://www.kobotoolbox.org/.

2 https://www.geoportal.gov.pl/pl/dane/baza-danych-obiektow-topograficznych-bdot10k/.
