# Supplementary material for: Community-based approach to detect and predict conflicts with large carnivores in human-dominated landscape
Source: Ambio. 2025 Sep 27;55(2):371–84. doi: 10.1007/s13280-025-02241-6 (PMC12779819; doi:10.1007/s13280-025-02241-6)
Supplement: Supplementary file 1 — Supplementary file1 (PDF 671 KB) [file 13280_2025_2241_MOESM1_ESM.pdf]

Supplementary Information

Izabela Fedyń[1,*], Marek Pasiniewicz[2], Katarzyna Zabiega[2], Hubert Fedyń[3], Michał Ciach[1]

Community-based approach to detect and predict conflicts with large carnivores in human-dominated landscape

[1] Department of Forest Biodiversity, Faculty of Forestry, University of Agriculture in Krakow, al. 29 Listopada 46, 31-425 Krakow, Poland

[2] Bieszczadziki Foundation, Bieszczadzka 106, 38-505 Bukowsko, Poland

[3] Regional Directorate for Environmental Protection in Rzeszów, ul. Józefa Piłsudskiego 38, 35-001, Rzeszów, Poland

[*]Corresponding author e-mail: izabela.fedyn@gmail.com

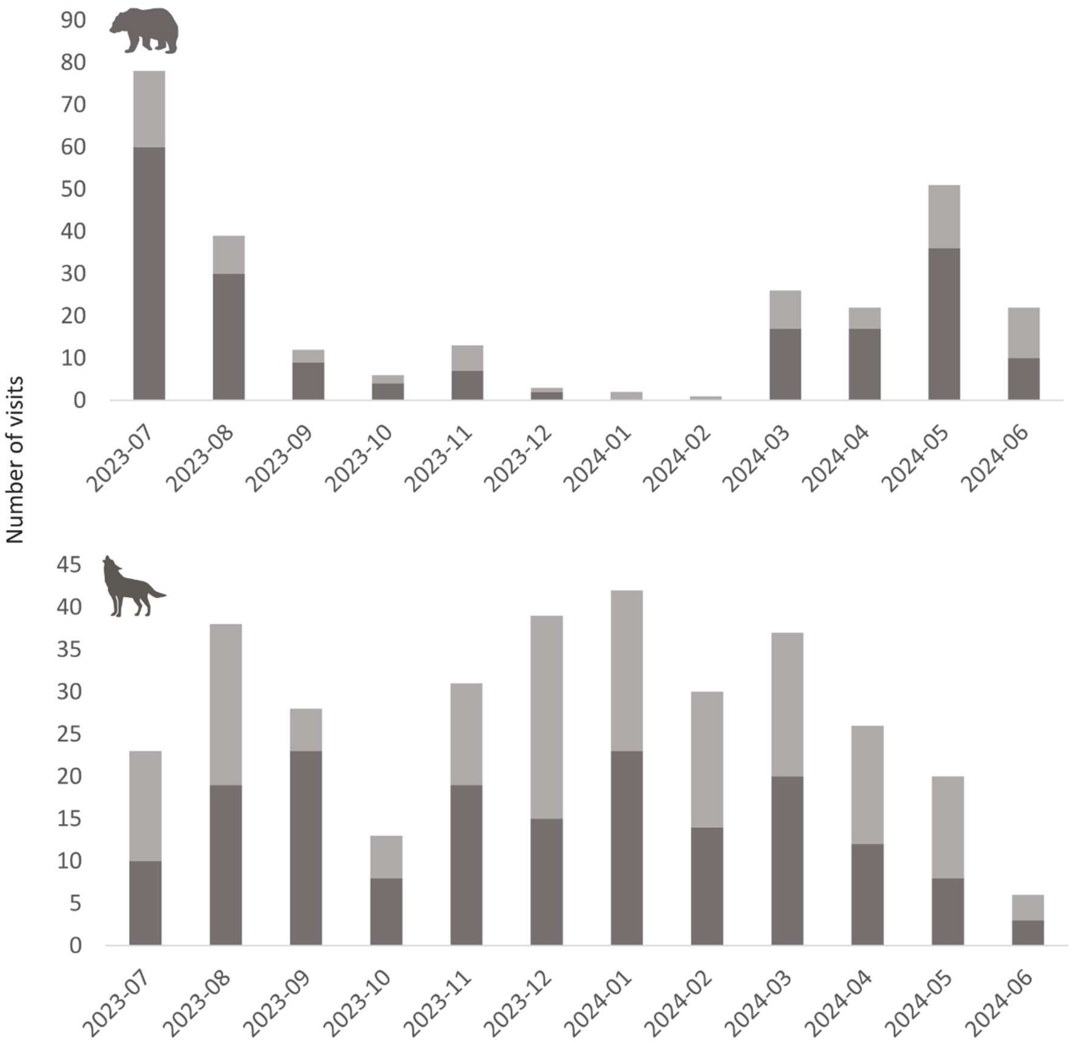

Supplementary Figure 1. Number of brown bear *Ursus arctos* (N = 277) and grey wolf *Canis lupus* (N = 334) records recorded by local communities in the Carpathians, southeastern Poland. The records are presented by month, with light gray bars indicating daytime visits and dark gray bars indicating nighttime visits.

Appendix 1. Form for community-reported large carnivore records.

A single event is defined as the activity of the same animal in the same place (or within 100 meters) within a time frame of up to 6 hours. If the animal appears again after this time or at a greater distance, it should be recorded as a separate event.

1. Date of the report
2. Time of the report
3. Geographic coordinates of the report

   Please mark the point on the map corresponding as precisely as possible to the reported observation location. For automatic positioning, the accuracy should be less than 20 meters.
4. Species involved

   Please choose one of the following:
   - Grey wolf
   - Brown bear
5. Number of animals observed

   Please indicate the minimum number of animals seen during this report
6. Animal behavior during observation

   Please select one or more of the following:
   - Moving
   - Foraging
   - Hunting/Attempting to acquire food
   - Observing surroundings
   - Resting
   - Social interactions (e.g. playing)
7. Observer's location and activity

   Please select one of the following:
   - No people at the event site (e.g. recorded by a camera)
   - Inside a vehicle (parked or moving)
   - Inside a building (e.g. observing through a window)
   - Outside, stationary (e.g. resting)
   - Walking on foot
   - Moving using sports equipment (e.g. bicycle, skis)
8. Potential attractants present

Were any of the following attractants recorded at the event site or nearby (up to 100 m) on the day of the event or up to a week earlier? Please select one or more of the following:

- Unsecured garbage (street bins, garbage bags, etc.)
- Plastic waste containers
- Metal waste containers
- Closed garbage shed
- Illegal dumpsite
- Compost heap
- Poultry
- Rabbits
- Dogs
- Sheep
- Goats
- Cattle
- Horses
- Farmed deer
- Wild ungulates (red deer, roe deer, etc.)
- Fruit shrubs (raspberries, blueberries, etc.)
- Fruit trees (apple, pear, plum, etc.)
- Wildlife feeding site
- Carcass
- Apiary
- None of the above
- Other

9. Damage caused by the animal

Did the observed animal cause any damage during the event? Please select one or more of the following:

- No
- Yes, property damage
- Yes, apiary destruction
- Yes, injury to an animal
- Yes, killing of an animal
- Yes, injury to a person

- Yes, killing of a person

10. Reporting and verification

    Please select one or more of the following:

    - Eyewitness report
    - Reports from two or more independent witnesses
    - Field verification by a trained person confirming the report (e.g. tracks observed)
    - Field verification by a trained person not confirming the report (no tracks found)
    - Photograph/Video
    - Report to the Regional Directorate for Environmental Protection (RDEP)
    - Police intervention at the event site

11. Photo documentation
12. Additional information
